# The effects of maturity matched and un-matched opposition on physical performance and spatial exploration behavior during youth basketball matches

**DOI:** 10.1371/journal.pone.0249739

**Published:** 2021-04-08

**Authors:** Jorge Arede, Sean Cumming, David Johnson, Nuno Leite

**Affiliations:** 1 Research Center in Sports Sciences, Health Sciences and Human Development, CIDESD, University of Trás-os-Montes and Alto Douro, Vila Real, Portugal; 2 Department for Health, University of Bath, Bath, United Kingdom; 3 A.F.C Bournemouth, Bournemouth, United Kingdom; Nottingham Trent University, UNITED KINGDOM

## Abstract

The aim of this study was analyze the effect of playing against biological matched and un-matched opposition, on physical performance and spatial exploration behavior of youth basketball players. Thirty under-14 to 16 basketball players were assigned to different teams according to maturity status (Pre-, Mid-, and Post-Peak Height Velocity [PHV]), and participated in basketball matches against matched (same maturity status), and un-matched (different maturity status) opposition. Maturity status was estimated considering the percentage of predicted adult height. Workload data was collected via inertial devices (IMUs) and Ultra-Wide Band (UWB)-based system. Heart rate was recorded with individual HR monitors. The Pre-PHV performed significantly more accelerations and decelerations and explored more space against matched opposition. Against un-matched opposition, the Pre-PHV presented higher average speed, body impacts, and Player Load. Both Mid- and Post-PHV covered more distance against matched opposition than against Pre-PHV. Games against Pre-PHV involved lower distance covered, average speed, Player Load, and higher accelerations and decelerations, than against Mid- and Post-PHV. The Pre-PHV athletes performed a higher number of accelerations and decelerations comparing to the Mid and Post-PHV players. Also, a significant interaction effect (group x time) was found in distance covered, average speed, body impacts, and Player Load. The type of opposition influenced physical performance and spatial exploration behavior during basketball matches, particularly of less-mature players. Based on present findings, practitioners can select the most suitable game format, considering the physical, technical, tactical, and psychological development needs, individualizing training stimulus.

## Introduction

Biological maturation refers to progress toward the adult or mature state and can be defined in terms of status, timing, and tempo [1]. Maturational status refers to the stage of maturation that an individual has attained (e.g., pre-pubertal, pubertal, post-pubertal); whereas tempo refers to the rate at which maturation progresses [1]. In contrast, the timing of maturation refers to the age at which specific maturational events (e.g., peak height velocity, pubertal onset, menarche) occur. Children of the same chronological age can demonstrate marked variance in the timing of maturation with some individual maturing well in advance or delay of their same age peers [1].

The timing of maturation has been shown to have significant impact upon the physical and athletic development of youth, and as a factor contributing to the selection and performance of young athletes [1,2]. Males who mature in advance of their peers, for example, tend to be taller, heavier, possess greater absolute and relative lean mass, and perform better on tests of strength, speed, and power. Accordingly, advanced maturation has been documented as an important predictor of performance [3] and a selection factor [4,5] in sports that prize such attributes, such as basketball. Maturity associated differences in anthropometrical, physical and psychological differences have been documented in youth basketball players [6]. Though players of advanced maturity status do not demonstrate marked advantages in sport-specific skills, such as dribbling, passing, shooting, and defensive movements [7], they do outperform less mature players in tests of speed, jump, agility, aerobic fitness, and throwing level [6]. Accordingly, early maturing male basketball players can be described as possessing both physical and athletic advantages [3,4]. Such advantages are, however, likely to be transient in nature as maturity associated differences in both size and function have shown to be attenuated, and in some cases reversed, in early adulthood [8].

Competition grouping strategies in team-sports like basketball, and soccer, have traditionally employed age-based criteria, using a “one-size-fits-all” approach, with limited consideration of individual differences in biological maturity. Bio-banding is an alternative athlete grouping strategy that has proposed to help counter some of the challenges presented by individual differences in growth and maturation [1,2,9,10]. In its simplest sense, bio-banding is described as the practice of grouping athletes relative to attributes associated with the processes of maturation (i.e., maturity matching), rather than age [1,2]. As a strategy, bio-banding seeks to attenuate maturity associated differences in size and function, encouraging players to emphasis and use their technical, tactical and psychological attributes [10,11]. Competing against older and more experienced players, early maturing youth are no longer able to rely on their physical advantages and must adapt their game, utilize their technical skills, think faster, and play as part of a team [10,11]. Late maturing players, in contrast, are more able to both use and demonstrate their physical, technical and tactical attributes, impact the game, and adopt positions of leadership [10,11].

Research investigating the practice of bio-banding and its potential benefits is limited to youth soccer [9,10,12]. To date, the results of these studies suggest that bio-banding benefits both early and late maturing players and changes the physical and technical demands of the game [9,10]. More recently, Abbott and colleagues [12] investigated the effect of bio-banding over chronological competition upon physical and technical performance in youth soccer athletes (85–90% of predicted adult stature). Results identified that bio-banded competition changed the technical demand placed upon athletes compared to the chronological competition, but no significant differences in physical performance were identified between competition formats [12]. In youth sports, bio-banding has most often been studied in football, but little is known about the effects and consequences of bio-banding in youth basketball.

Arede and colleagues [13] were the first to examine differences in external and internal load between traditional (i.e. chronological age) and bio-banded competitions in youth basketball. The results confirmed that the players covered more distance, in jogging (>6.0–12.0 km∙h^-1^), running (>12.10–18.0 km∙h^-1^) during traditional competition over bio-banding [13]. Also, a significantly higher number of lower body impacts (> 5g) and Player load [13] were observed in the bio-banded competition [13]. Nevertheless, further research is needed to extrapolate which variables, including external, and internal load, and individual exploration behavior, are interchangeable in the bio-banding approach. Considering the previous statements and the need to provide some recommendations to support coaches how to recognize differences in maturation level, and use the bio-banding approach to individualize training, this research study aimed to analyze the effect of playing against biological matched and un-matched opposition, on physical performance and spatial exploration behavior of youth basketball players. More specifically, we sought to examine how parameters of physical performances varied when athletes competed in game formats when they were matched or not matched by maturational status.

## Materials and methods

### Experimental design

Using a descriptive design, all data were gathered over 2 sessions with a 48-h interval, involving matched and un-matched game formats. Due to equipment availability, only eighteen players (six per maturity band were randomly selected) were monitored. Players’ movement demands were assessed using the Ultra-Wide Band (UWB)-based system (*Realtrack Systems*, *Almeria*, *Spain*). Physical performance tests were conducted one week before the commencement of the study, as described elsewhere [5,14] for better understanding of the influence of somatic maturation on physical parameters among sample subjects.

### Participants

Eighteen under-14 and twelve under-16 national level basketball players (age = 13.45±1.22 years; height = 164.52±11.85 cm; body mass = 55.90±12.84 kg; percentage of their predicted adult height [% PAH] = 90.75±5.40%) were recruited from Portuguese Basketball Academy to participate in this study. All players averaged a total of six hours of basketball training (4 team-sessions/week, 90 minutes/session), and 1 competitive match (national level) per week. Written informed consent was obtained from all participants and their parents before this investigation. The present study was approved by the Ethics Committee of the University of Trás-os-Montes and Alto Douro and conformed to the recommendations of the Declaration of Helsinki.

### Somatic maturation

Height was recorded using a commercial portable stadiometer (Tanita BF-522W, Japan, nearest 0.1 cm). Body mass was estimated using the scales (Tanita BF-522W, Japan, nearest 0.1 kg). All measurements were taken following the guidelines outlined by the *International Society for the Advancement of Kinanthropometry* (ISAK) by the same researcher, who holds an ISAK Level 1 accreditation. Players’ height, weight, chronological age, and mid-parent height were used to predict the adult height of each player [15]. The height of the biological parents of each player were self-reported and adjusted for over-estimation using the previously established equations [16]. The current height of each player was then expressed as a percentage of their predicted adult height (% PAH), which can then be used as an index of somatic maturation [17]. Players were grouped into three maturity bands based on the percentage of predicted adult height attained at the time of the tournament [2]: <88% (Pre-PHV), 88–95% (Mid-PHV) and >95% (Post-PHV) of predicted adult stature (Table 1). Only for descriptive reasons, maturity timing was estimated for each player based on z-scores: average or on-time (z-score between +0.5 and -0.5), early (z-score >+0.5), and late (z-score <-0.5) [9].

**Table 1:** Participant characteristics of maturation groups.

Maturity band	Pre-PHV (n = 10)	Mid-PHV (n = 10)	Post-PHV (n = 10)	Effect Sizes		
				Pre-PHV vs. Mid-PHV ^a^^,^^b^	Pre-PHV vs. Post-PHV ^a^^,^^b^	Mid-PHV vs. Post-PHV ^a^^,^^b^
Age (years)	12.14 ± 0.74 (11.6–13.5)	13.53 ± 0.63 (12.3–14.4)	14.69 ± 0.46 (14.3–15.9)	-2.03 ^b^	-4.16 ^b^	-2.10 ^b^
PAH (%)	84.05 ± 2.39	92.11 ± 1.82	96.09 ± 1.06	-3.79 ^b^	-6.51 ^b^	-2.68 ^b^
Maturity Timing	Early = 4	Early = 8	Early = 8			
	On-time = 3	On-time = 2	On-time = 2			
	Late = 3	Late = 0	Late = 0			
Height (cm)	149.61 ± 5.60	171.55 ± 5.20	172.40 ± 4.79	-4.06 ^b^	-4.37 ^b^	-0.17
Weight (kg)	43.64 ± 8.36	59.10 ± 10.68	64.96 ± 8.83	-1.61 ^b^	-2.48 ^b^	-0.60
Abalakov Jump (cm)	30.00 ± 4.45	36.03 ± 6.79	43.14 ± 3.07	-1.05 ^a^	-3.44 ^b^	-1.35 ^b^
V-cut test (s)	8.25 ± 0.14	7.75 ± 0.31	7.34 ± 0.38	-2.11 ^b^	-3.17 ^b^	-1.18 ^a^
0–25 sprint time (s)	3.82 ± 0.14	3.41 ± 0.24	3.18 ± 0.14	-2.08 ^b^	-4.54 ^b^	-1.17 ^a^

PAH, Predicted adult height.

^a^ Significant difference (*p*< 0.05);

^b^ Significant difference (*p*< 0.01).

### Game formats

**Fig 1:**
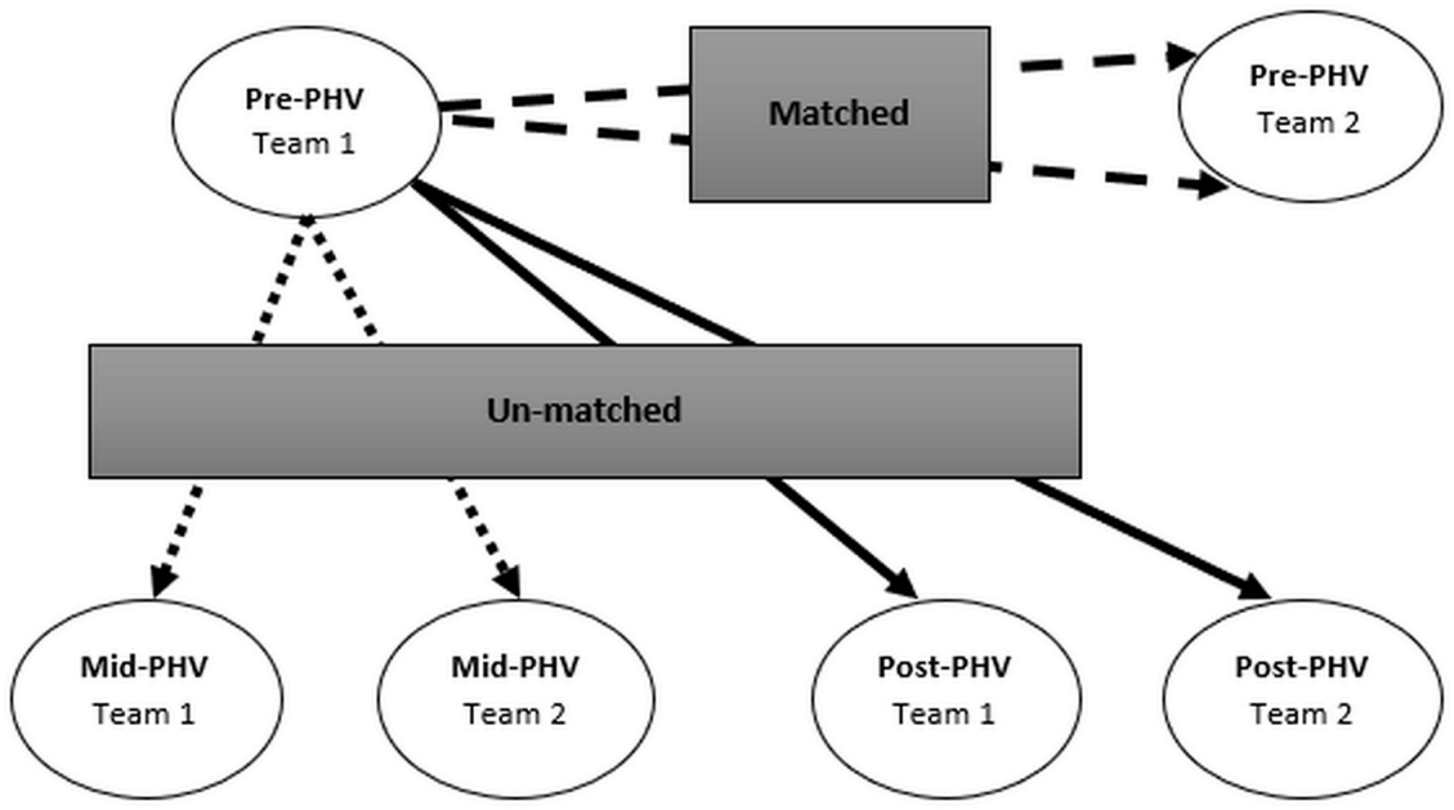
Schematic representation of game situations (Pre-PHV example).

Subjects were included in one of two teams per maturity band. Participants competed in maturity matched (i.e., bio-banded) and non-matched (i.e., different bio-bands) formats against external opposition that had also been grouped in three equivalent maturity bands (Fig 1). Each team competed twice against each maturity band. In total, each participant completed a total of 6 games (Fig 1). Games were played in 5 vs. 5 formats, with 8-min duration, and conformed to standard officiating and rule procedures. Matches were played on a full-sized standard basketball court (28 × 15 m), with full-sized baskets (3.05 m), with a size 7-ball. Games were played without substitutions, verbal encouragement and technical or tactical instructions (including time-outs). Subjects were instructed to stay at the basketball field during the game [18]. Moreover, the main researcher provided new balls when necessary to ensure the quick restart of game situations [19], and called any foul or rules violation.

### Physical and tactical data

The physical and tactical data was collected using inertial devices (IMUs) and Ultra-Wide Band (UWB)-based system placed in indoor basketball court (WIMU PRO^®^; *Realtrack Systems*, *Almeria*, *Spain*). IMUs integrates multiple sensors registering at different sample frequencies [20]. For example, sampling frequency for a 3-axis accelerometer, gyroscope, and magnetometer was 100 Hz, 120 kPa for the barometer, and 18 Hz for the positioning system [20]. The WIMU PRO^®^ showed satisfactory accuracy (x-axis = 5.2 ± 3.1 cm; y-axis 5.8 ± 2.3 cm) and reliability (x-axis, ICC = 0.65; y-axis, ICC = 0.85) in fixed reference lines of a basketball court at a speed of over 15 km/h, [21]. The same system showed better accuracy (bias: 0.57–5.85%), test–retest reliability (%TEM: 1.19), and inter-unit reliability (bias: 0.18) in determining distance covered and mean velocity (bias: 0.09; ICC: 0.979; bias: 0.01 > bias: 0.18; ICC: 0.951; bias: 0.03) than GPS technology [22]. The UWB system was composed of 6 antennae placed in a hexagon around the indoor basketball court [23,24]. The UWB system uses triangulations between the antennas and the inertial units, six antennas send a signal to the units every 50 milliseconds [24]. Then, the device calculates the time required to receive the signal and derives the unit position (coordinates X and Y), using one of the antennas as a reference [24]. The use of inertial devices and calibration process occurred as described elsewhere [23]. The following variables were calculated per minute: (a) distance covered (DC; m), (b) DC at ≤ 6 km∙h^-1^ (speed zone 1 [SZ1]), (c) DC from 6.1 to 12 km∙h^-1^ (speed zone 2 [SZ2]), (d) DC at > 18 km∙h^-1^ (speed zone 3 [SZ3]), (e) high-intensity accelerations (HIAcc) and decelerations (HIDec), (f) Body impacts (BI >5G), and (g) Player load (PL). Also, average and peak speed (km∙h^-1^) peak acceleration (PAcc; m∙s^-2^) and deceleration (PDec; m∙s^-2^) were calculated [20]. Speed zones during the basketball game were adapted from a previous research [24]. The > 2 m∙s^-2^ and > -2 m∙s^-2^ was the criteria to detect high-intensity accelerations and decelerations, respectively [25]. Body impacts correspond to the number of jumps and impacts that exceed 5G forces, measured with the inertial accelerometer in the z, x, and y axes [26]. The instantaneous Player load (PLn) was computed using the following formula: $PLn=\frac{\sqrt{{\left(Xn-Xn-1\right)}^{2}+{\left(Yn-Yn-1\right)}^{2}+{\left(Zn-Zn-1\right)}^{2}}}{100}$. The accumulated Player Load (PL) $(PL=\sum_{n=0}^{m}\left(PLn)\;x\;0,01\right)$ was computed and calculated per minute for further analysis.

The heart rate (HR) data were recorded continuously with individual HR monitors (*Garmin*, *Soft Strap Premium*, *USA*). Average and participants’ peak HR (HR peak), taken as the highest HR recorded throughout the testing period [27] were recorded. The HR peak zones were defined as: zone 1 (50–60%); zone 2 (60–70%); zone 3 (70–80%); zone 4 (80–90%), and zone 5 (90–100%). The Edward’s training impulses (TRIMP) was calculated based on the following formula and presented in arbitrary unit (a.u.) [28]: TRIMP = (time spent in zone 1 * 1) + (time spent in zone 2 * 2) + (time spent in zone 3 * 3) + (time spent in zone 4 * 4) + (time spent in zone 5 * 5).

Sample Entropy (SampEn) was also used to assess each player’s HR regularity during the games. SampEn (m,r,n) is defined as the negative natural logarithm of the conditional probability that two sequences, similar form points (length of the vector to be compared), remain similar at the next point m + 1 [29] The values used to calculate SampEn were 2 to vector length (m) and 0.2 ±SD to the tolerance (r) [29]. Values of SampEn range from zero towards infinity, where values close to zero were indicative of higher regularity in HR, while the higher the SampEn, the more unpredictable the HR. All data were analyzed using the commercially available software *(WIMU SPRO Software; Realtrack Systems SL*). Spatial exploration index (SEI) was obtained for each player by calculating his mean pitch position, computing the distance from each positioning time-series to the mean position and, finally, computing the mean value from all the obtained distances [30].

### Statistical analyses

Data are presented as mean ± SD. All data was found to be normally distributed using the Shapiro-Wilk test. Univariate analysis of variance (ANOVA) test and Tukey’s post-hoc test were used in conjunction to examine the differences between groups (Pre-, Mid-, and Post-PHV), in all parameters. Effect sizes (ES) of the differences between and within-groups were evaluated using the Cohen’s “*d*”. Also, repeated-measures ANOVA was used to analyze within-group changes. A 3x3 repeated-measures ANOVA was performed on the absolute values of all parameters to determine the main effects between groups (Pre-, Mid-, and Post-PHV) and game formats (Pre-, Mid-, and Post-PHV). Sphericity of the data was checked by Mauchly’s statistic, and where violated, Greenhouse-Geiser adjustment was applied. The level of statistical significance was set at *p* ≤ 0.05. All the statistical analyses were performed using SPSS software (version 24 for Windows; *SPSS Inc*., *Chicago*, *IL*, *USA*). All graphs were constructed in computing environment R (Version 1.2.1335, RStudio, 2019), using ggplot2 package [31].

## Results

**Fig 2:**
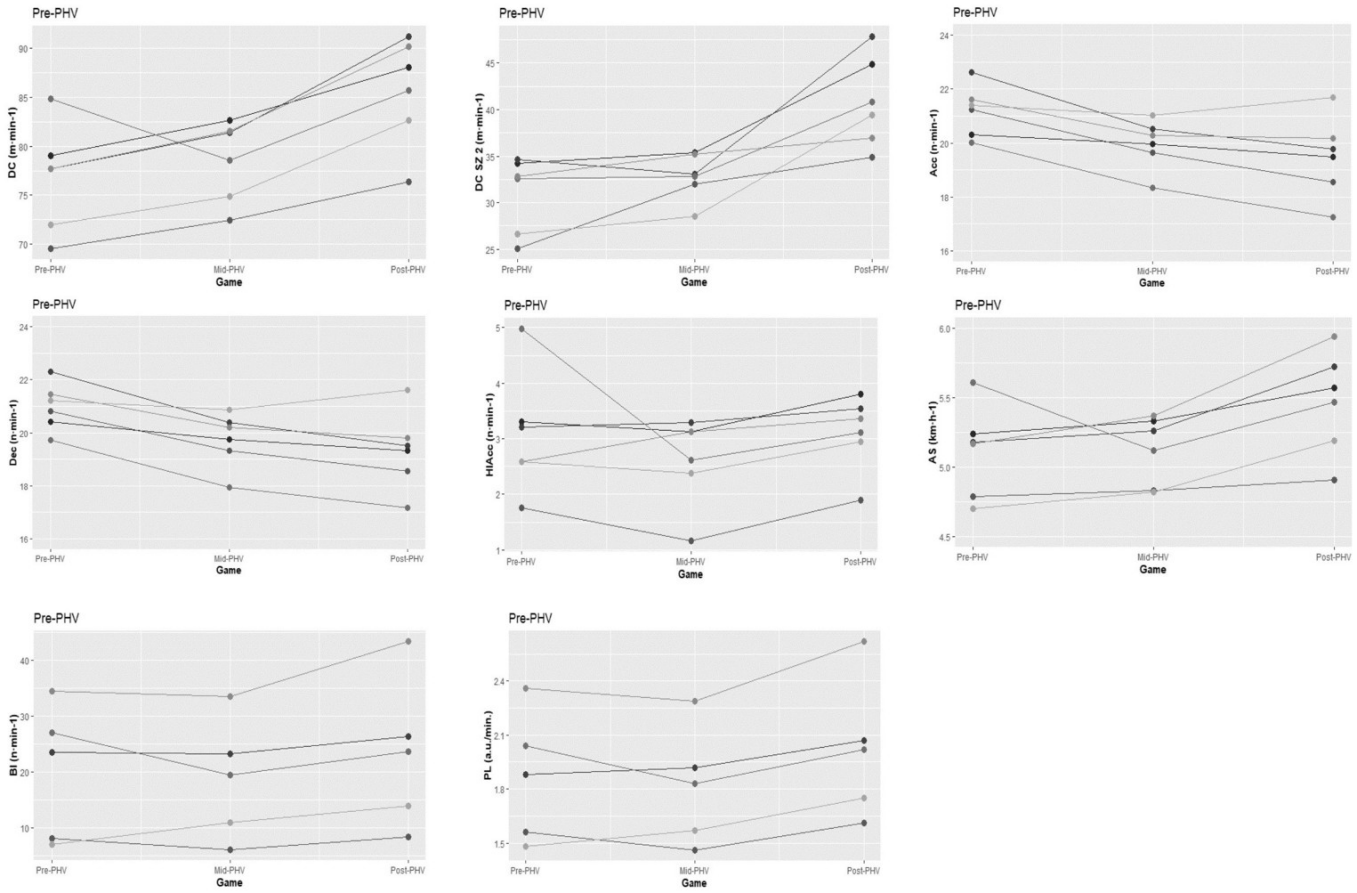
Individual graphical representation of significant differences in physical and tactical performance between game formats amongst Pre-PHV athletes.

**Fig 3:**
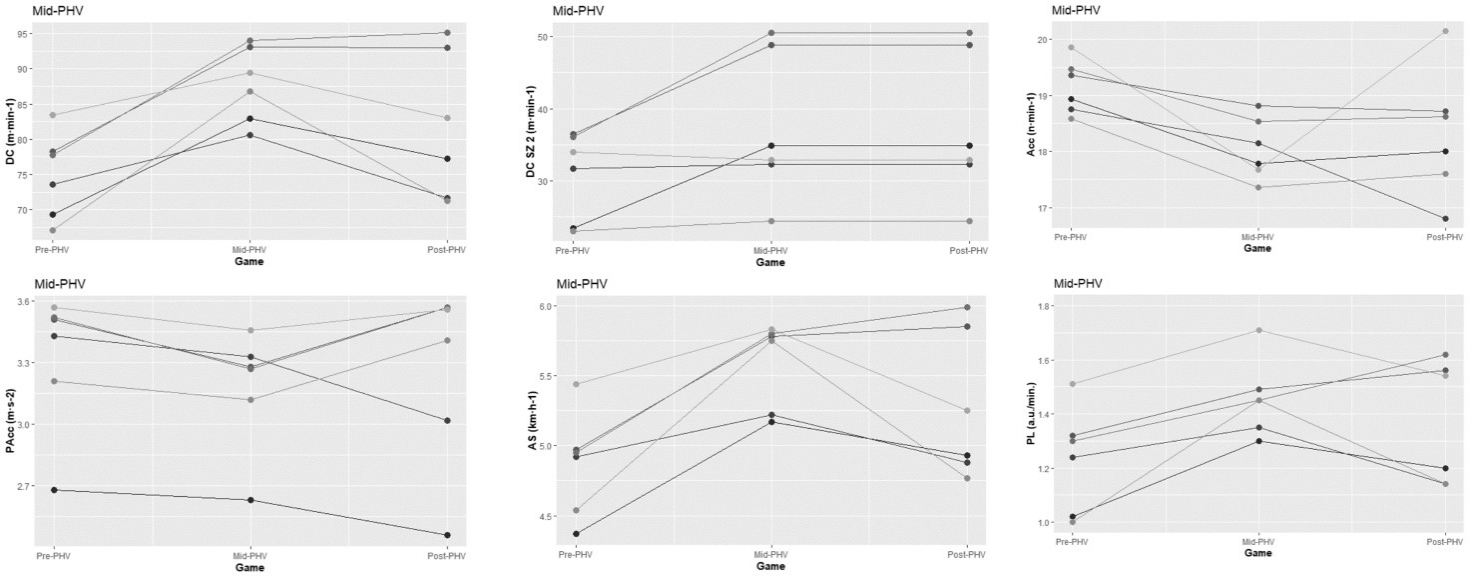
Individual graphical representation of significant differences in physical and tactical performance between game formats amongst Mid-PHV athletes.

**Fig 4:**
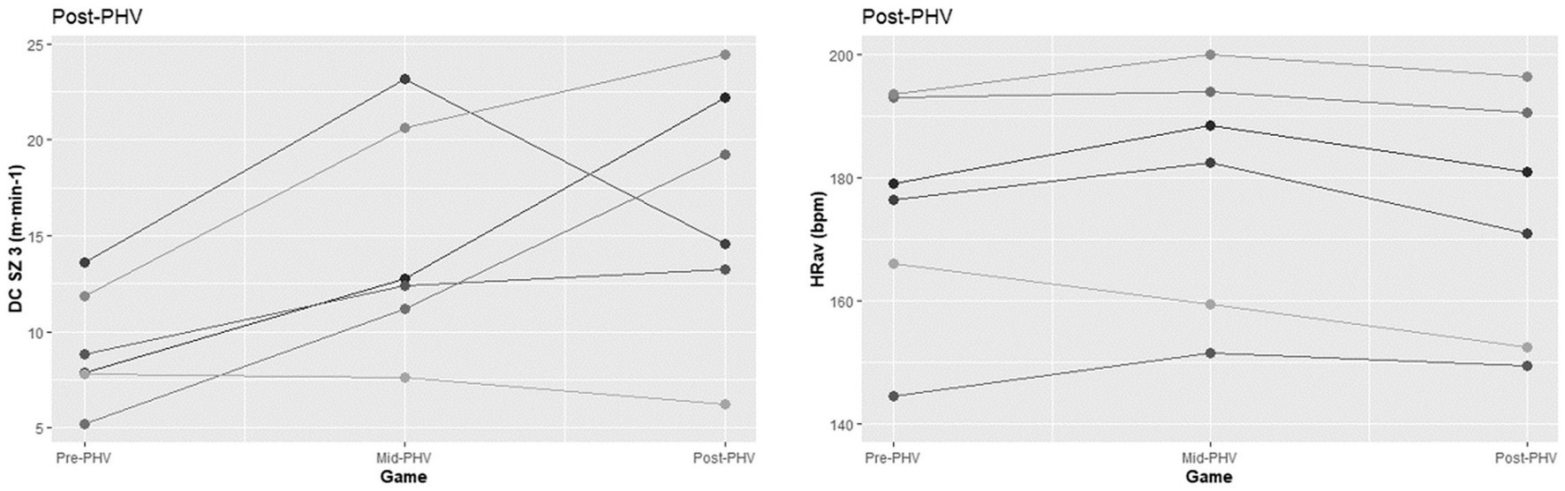
Individual graphical representation of significant differences in physical and tactical performance between game formats amongst Post-PHV athletes.

The Pre-PHV teams covered greater distances when competing against Post-PHV teams than against Pre-PHV (*p < 0*.*05*) and Mid-PHV (*p < 0*.*01*) teams (Table 2; Fig 2). The Pre-PHV teams also performed more accelerations (*p < 0*.*05*) and decelerations (*p < 0*.*05)* when competing against matched opposition than against the Mid-PHV. Furthermore, the Pre-PHV teams presented higher mean values for AS (*p < 0*.*05*), BI (*p < 0*.*05*), and PL (*p < 0*.*01*) when competing against un-matched opposition (Table 2; Fig 2). The Pre-PHV obtained higher mean values for SEI when competing in maturity matched games than when competing against un-matched opposition (all *p < 0*.*05*) (Table 2; Fig 2). The Mid-PHV accumulated higher DC (*p < 0*.*05*), performed more HIAcc (*p < 0*.*05*), shown higher AS (*p < 0*.*01*), and PL (*p < 0*.*05*) playing against matched opposition than against Pre-PHV (Table 3; Fig 3). Finally, the Post-PHV presented higher DC when played against matched opposition than against Pre-PHV (*p < 0*.*05*), but also lower AS when played against Pre-PHV than against Mid-PHV or Post-PHV (Table 4; Fig 4).

**Table 2:** Mean (± SD) physical and tactical performance produced by Pre-PHV during game formats.

Variables	Matched (vs. Pre-PHV)	Non-Matched (vs. Mid-PHV)	Non-Matched (vs. Post-PHV)	Effect Sizes		
				Matched vs. Non-Matched (Mid-PHV) ^a^^,^^b^	Matched vs. Non-Matched (Post-PHV) ^a^^,^^b^	Non-Matched (Mid-PHV) vs. Non-Matched (Post-PHV) ^a^^,^^b^
DC (m∙min^-1^)	76.80 ± 2.22	78.57 ± 1.68	85.68 ± 2.26	-0.45	-1.93 ^a^	-3.33 ^a^
DC SZ 1 (m∙min^-1^)	32.16 ± 3.26	33.69 ± 2.61	32.52 ± 1.89	-1.34	-0.14	0.59
DC SZ 2 (m∙min^-1^)	30.98 ± 4.10	32.85 ± 2.51	40.78 ± 4.85	-0.65	-2.92 ^b^	-1.61 ^a^
DC SZ 3 (m∙min^-1^)	13.67 ± 4.94	12.04 ± 4.41	12.39 ± 5.73	0.32	0.20	-0.07
Acc (n∙min^-1^)	21.19 ± 0.94	19.96 ± 0.93	19.49 ± 1.50	1.71 ^a^	1.34	0.71
Dec (n∙min^-1^)	20.99 ± 0.90	19.75 ± 1.03	19.33 ± 1.47	2.02 ^a^	1.40	0.69
HIAcc (n∙min^-1^)	3.07 ± 1.09	2.62 ± 0.79	3.12 ± 0.67	0.45	-0.04	-2.34 ^b^
HIDec (n∙min^-1^)	2.80 ± 1.10	2.54 ± 0.98	3.10 ± 0.95	0.24	-0.25	-0.90
PAcc (m∙s^-2^)	3.57 ± 0.31	3.54 ± 0.34	3.56 ± 0.25	0.19	0.02	-0.19
PDec (m∙s^-2^)	-3.86 ± 0.65	-3.63 ± 0.35	-3.59 ± 0.38	0.41	0.49	0.23
AS (km∙h^-1^)	5.11 ± 0.33	5.12 ± 0.24	5.46 ± 0.37	-0.03	-1.07	-2.00 ^a^
PS (km∙h^-1^)	18.33 ± 1.78	18.05 ± 1.76	17.10 ± 1.92	0.52	1.35	0.99
BI (n∙min^-1^)	20.67 ± 10.86	19.45 ± 9.80	23.71 ± 12.13	0.33	-0.69	-1.52 ^a^
PL (a.u./min.)	1.87 ± 0.32	1.83 ± 0.29	2.02 ± 0.35	0.31	-1.36	-2.74 ^b^
HR_av_ (bpm)	185.67 ± 3.72	184.00 ± 4.94	187.20 ± 3.49	0.31	-0.51	-1.21
HR_SampEn_ (a.u.)	0.54 ± 0.13	0.50 ± 0.20	0.50 ± 0.20	0.22	0.17	-0.00
TRIMP (a.u.)	34.30 ± 3.60	35.46 ± 1.70	35.95 ± 1.80	-0.45	-0.77	-0.31
SEI (a.u.)	6.58 ± 0.49	5.97 ± 0.66	6.09 ± 0.58	1.10	1.12	-0.42

DC, distance covered; SZ1, speed zone 1; SZ2, speed zone 2; SZ3, speed zone 3; Acc, accelerations; Dec, decelerations; HIAcc, high-intensity accelerations; HIDec, high-intensity decelerations; PAcc, peak acceleration; PDec, peak deceleration; AS, average speed; PS, peak speed; BI, body impacts (> 5g); PL, player load; HR_av_, average heart rate; HR_SampEn_, heart rate sample entropy TRIMP, training impulse; SEI, spatial exploration index.

^a^ Significant difference (*p*< 0.05);

^b^ Significant difference (*p*< 0.01).

**Table 3:** Mean (± SD) physical and tactical performance produced by Mid-PHV during game formats.

Variables	Non-Matched (vs. Pre-PHV)	Matched (vs. Mid-PHV)	Non-Matched (vs. Post-PHV)	Effect Sizes		
				Matched vs. Non-Matched (Pre-PHV) ^a^^,^^b^	Matched vs. Non-Matched (Post-PHV) ^a^^,^^b^	Non-Matched (Pre-PHV) vs. Non-Matched (Post-PHV) ^a^^,^^b^
DC (m∙min^-1^)	74.91 ± 6.12	87.81 ± 5.40	81.88 ± 10.39	2.39 ^b^	0.97	-0.88
DC SZ 1 (m∙min^-1^)	35.99 ± 3.88	34.96 ± 3.31	33.88 ± 4.30	-0.54	0.38	0.55
DC SZ 2 (m∙min^-1^)	30.78 ± 6.08	39.03 ± 4.38	37.31 ± 10.23	1.65 ^a^	0.28	-0.94
DC SZ 3 (m∙min^-1^)	8.15 ± 3.53	13.82 ± 5.88	10.70 ± 4.09	1.20	0.88	-0.53
Acc (n∙min^-1^)	19.16 ± 0.49	18.05 ± 0.55	18.31 ± 1.14	-1.85 ^a^	-0.21	1.17
Dec (n∙min^-1^)	19.01 ± 0.49	17.84 ± 0.76	18.29 ± 1.17	-1.26	-0.31	1.05
HIAcc (n∙min^-1^)	2.01 ± 0.71	2.82 ± 0.80	2.04 ± 0.78	1.10	1.21	-0.05
HIDec (n∙min^-1^)	1.58 ± 0.75	1.62 ± 0.53	1.68 ± 0.78	0.10	-0.08	-0.22
PAcc (m∙s^-2^)	3.32 ± 0.34	3.18 ± 0.29	3.27 ± 0.45	-1.70 ^a^	-0.31	0.26
PDec (m∙s^-2^)	-3.23 ± 0.38	-3.20 ± 0.39	-3.24 ±0.52	-0.10	-0.19	-0.04
AS (km∙h^-1^)	4.86 ± 0.37	5.59 ± 0.31	5.28 ± 0.52	2.18 ^b^	0.73	-0.83
PS (km∙h^-1^)	17.49 ± 0.85	17.59 ± 0.99	17.11 ± 0.82	0.34	1.13	0.77
BI (n∙min^-1^)	6.04 ± 4.27	6.74 ± 3.53	6.83 ± 4.61	0.59	-0.05	-0.65
PL (a.u./min.)	1.23 ± 0.19	1.46 ± 0.14	1.36 ± 0.23	1.83 ^a^	0.51	-0.86
HR_av_ (bpm)	167.63 ± 15.00	170.88 ± 9.56	169.71 ± 14.74	0.54	0.19	-0.37
HR_SampEn_ (a.u.)	0.30 ± 0.20	0.45 ± 0.08	0.41 ± 0.12	1.06	0.50	-0.67
TRIMP (a.u.)	29.33 ± 5.03	31.69 ± 3.11	30.23 ± 4.60	1.02	0.73	-0.38
SEI (a.u.)	6.22 ± 0.22	5.95 ± 0.27	6.25 ± 0.64	-1.14	-0.58	-0.06

DC, distance covered; SZ1, speed zone 1; SZ2, speed zone 2; SZ3, speed zone 3; Acc, accelerations; Dec, decelerations; HIAcc, high-intensity accelerations; HIDec, high-intensity decelerations; PAcc, peak acceleration; PDec, peak deceleration; AS, average speed; PS, peak speed; BI, body impacts (> 5g); PL, player load; HR_av_, average heart rate; HR_SampEn_, heart rate sample entropy TRIMP, training impulse; SEI, spatial exploration index.

^a^ Significant difference (*p*< 0.05);

^b^ Significant difference (*p*< 0.01).

**Table 4:** Mean (± SD) physical and tactical performance produced by Post-PHV during game formats.

Variables	Non-Matched (vs. Pre-PHV)	Non-Matched (vs. Mid-PHV)	Matched (vs. Post-PHV)	Effect Sizes		
				Matched vs. Non-Matched (Pre-PHV) ^a^^,^^b^	Matched vs. Non-Matched (Mid-PHV) ^a^^,^^b^	Non-Matched (Pre-PHV) vs. Non-Matched (Mid-PHV) ^a^^,^^b^
DC (m∙min^-1^)	77.00 ± 8.05	81.26 ± 11.57	84.67 ± 9.39	1.41	0.62	-0.97
DC SZ 1 (m∙min^-1^)	37.02 ± 2.86	33.53 ± 1.92	33.56 ± 3.79	-0.62	0.01	0.91
DC SZ 2 (m∙min^-1^)	30.81 ± 7.29	33.10 ± 7.01	34.46 ± 7.22	1.26	0.66	-0.46
DC SZ 3 (m∙min^-1^)	9.18 ± 3.04	14.64 ± 5.97	16.65 ± 6.67	0.31	1.06	-1.53 ^a^
Acc (n∙min^-1^)	19.20 ± 0.49	18.70 ± 0.74	18.01 ± 2.03	-0.40	-0.56	0.70
Dec (n∙min^-1^)	19.15 ± 0.52	18.53 ± 0.81	17.84 ± 1.89	-0.43	-0.64	0.74
HIAcc (n∙min^-1^)	2.15 ± 0.43	2.28 ± 0.81	2.40 ± 0.68	0.25	0.32	-0.17
HIDec (n∙min^-1^)	2.14 ± 0.56	2.27 ± 0.78	2.37 ± 0.72	0.48	0.20	-0.24
PAcc (m∙s^-2^)	3.38 ± 0.23	3.38 ± 0.38	3.36 ± 0.22	-0.10	-0.18	-0.02
PDec (m∙s^-2^)	-3.54 ± 0.29	-3.39 ± 0.37	-3.55 ± 0.28	-0.13	-1.02	-0.76
AS (km∙h^-1^)	4.92 ± 0.39	5.25 ± 0.57	5.46 ± 0.54	1.40	0.65	-1.20
PS (km∙h^-1^)	18.05 ± 0.68	18.27 ± 0.68	17.92 ± 0.54	-0.20	-0.32	-0.25
BI (n∙min^-1^)	9.41 ± 3.77	12.32 ± 6.19	11.51 ± 5.14	0.83	-0.43	-0.81
PL (a.u./min.)	1.42 ± 0.27	1.59 ± 0.45	1.57 ± 0.35	-0.11	1.20	-0.80
HR_av_ (bpm)	175.42 ± 18.41	179.33 ± 19.52	173.50 ± 19.48	-0.28	-1.66 ^a^	-0.67
HR_SampEn_ (a.u.)	0.40 ± 0.12	0.51 ± 0.13	0.51 ± 0.08	0.83	0.03	-0.69
TRIMP (a.u.)	28.92 ± 5.50	29.35 ± 9.14	29.02 ± 5.56	0.02	-0.06	-0.06
SEI (a.u.)	6.16 ± 0.73	5.68 ± 0.55	5.71 ± 0.33	-0.58	0.04	0.62

DC, distance covered; SZ1, speed zone 1; SZ2, speed zone 2; SZ3, speed zone 3; Acc, accelerations; Dec, decelerations; HIAcc, high-intensity accelerations; HIDec, high-intensity decelerations; PAcc, peak acceleration; PDec, peak deceleration; AS, average speed; PS, peak speed; BI, body impacts (> 5g); PL, player load; HR_av_, average heart rate; HR_SampEn_, heart rate sample entropy TRIMP, training impulse; SEI, spatial exploration index.

^a^ Significant difference (*p*< 0.05);

^b^ Significant difference (*p*< 0.01).

Inferential analysis also confirmed a significant main effect of time in DC, DC in SZ2, Acc, Dec, AS, PS, PL, and SEI (all *p* < 0.01). Games involving Pre-PHV involved lower DC (Mid and Post-PHV), DC in SZ2 (Mid and Post-PHV), AS (Mid and Post-PHV), BI (Post-PHV), and PL (Mid and Post-PHV), but also higher Acc and Dec (Mid and Post-PHV), PS (Post-PHV), and SEI (Mid-PHV). Games involving Mid-PHV involved lower DC in SZ2, but also higher PS comparing to those involving Post-PHV.

A significant effect of group was found in Acc, Dec, BI, and PL (all *p* < 0.05). The Pre-PHV athletes performed more Acc and Dec comparing to the Mid and Post-PHV players. Moreover, performed higher values of HIDec, BI, PL than Mid-PHV. Also, a significant interaction effect (group x time) was found in DC, DC in SZ2, AS, BI, and PL (all *p* < 0.05).

## Discussion

The purpose of this investigation was to examine the impact of maturity matching upon physical performance and spatial exploration behavior in youth basketball players. As expected, the degree to which players were matched or not matched, influenced the tactical complexity and physical demands of the games. The impact of maturity matching was most pronounced in less mature (i.e., Pre-PHV) players who appeared to benefit the most from competing with maturity matched peers.

In the present study, the internal load remained constant irrespective of game formats. Pre-, Mid-, and Post-PHV players present the physical effort equal regardless of whether they were competing against maturity matched or unmatched opposition. These findings are consistent with previous study which revealed similar internal load (maximum and HR_av_), irrespective of the competition format (chronological vs bio-banding) [13]. The absence of differences in the internal load may reflect limited impact of maturation status upon aerobic capacity. For example, laboratory studies demonstrated no significant main effect of maturation status (Pre-, Mid-, and Post- Pubertal) in HR response during submaximal cycling exercise 30 to 70% of peak oxygen uptake [32].

The games involving the Pre-PHV teams (Pre- vs Pre-PHV; Pre- vs Mid-PHV; and Pre-PHV vs. Post-PHV) were characterized by a decrease in total distance covered. That is, all players covered less distances when competing in games that involved Pre-PHV players. The lower distances covered by Mid- and Post-PHV players against Pre-PHV players may result from greater full-court pressure which generated more opportunities to tackle and steal the ball, and perform more effective shots closer to the basket [33,34]. Furthermore, in maturity mismatched competition, steals are extensively performed by heavier, taller, and more mature players [3,4]. Thus, when competing against their less mature peers, the more mature players (i.e., Post-PHV) in the current study, may have used their physical advantage to score more points, without having to run such long distances. Afterwards, it is difficult to explain lower distance in Pre-PHV matched game based on previous arguments, and more studies are need for better understanding.

The results of analysis pertaining spatial exploration were of particular interest. More specifically, Pre-PHV players presented higher mean values for individual spatial exploration behavior (i.e., SEI) when competing in maturity matched games. This indicates that Pre-PHV players adjusted their tactical behaviors according to the maturational status of the opponents. The SEI has been shown to be highly dependent on environmental constraints such as the amount space available to play [30]. The physical and functional advantages associated with variance in maturity may equally serve as important task-based constraint, impacting spatial exploration in youth basketball [35]. According to previous findings, players tend to explore more space when they play without restriction in space occupation [30], but also against lower anthropometrical disadvantage [35]. When Pre-PHV players played against matched opposition probably feel freer to move through the space [10]. It seems likely that anthropometrical differences could generate a distinct perception of free space available, underpinning different individual exploration behavior. The concern about the anthropometric aspects, the pitch size, and the spatial awareness has been addressed in previous research exploring bio-banding in youth football [9]. In all game formats, from 6-a-side to 11-a-side, less mature players (80–85% PAH) play in smaller pitch sizes comparing to more mature players (91–95% PAH) [9]. This adjustment reflects one advance comparing with previous bio-banding experience where players played in standard pitch irrespective of their biological maturation [10]. Thus, future research could examine how the variation of available space and anthropometric aspects may affect spatiotemporal interpretation in youth athletes. Moreover, the game involving matched opposition imposes longer offensive sequences, a greater number of passes [12], and more displacements in lateral and longitudinal directions [36] to find a solution to complete the offensive phase. In contrast, Pre-PHV against non-matched opposition significantly explored less space, in line with previous findings [35]. Against un-matched maturity opposition, players need to execute faster than they normally would [10], and they had less time for better interpretation of spatiotemporal information [35]. However, against matched opposition, there is greater physical equity [1,9,10,12] and therefore the differences in size and strength are likely to have a greater impact on performance, and less mature players seem less reluctant to perform their ball or non-ball skills.

Player Load and the body impacts are frequently used to quantify the external training load during basketball training and matches [13,20,28]. According to the present findings, the games involving the Pre-PHV resulted in lower values of Player Load and body impacts. Although in youth basketball more mature players are frequently involved in actions that involve physicality [14], when competing against their less mature peers, they play less physical and competitive intensity. Matched groups increase the opportunities to work with right balance between competence and challenge (i.e. zone of proximal development), which is beneficial for psychological development [37]. In this regard, playing against matched opposition resulting in higher level of enjoyment and pleasantly, but also higher physical and technical challenge [9]. However, if more mature players are given a decreased challenge (e.g., players with decreased physical attributes) then they will be less likely to try hard, which may result in lower values of Player Load and body impacts.

Considering the lower values of Player Load and body impacts, playing against un-matched opposition may be especially useful when players are going through developmental phases in which they may be more susceptible to injury risk (i.e., the pubertal growth spurt) [11], because it imposes lower external demands. Nevertheless, Pre-PHV faced higher inter-game variability and absolute values (against Post-PHV) in terms of PL and BI, resulting in a higher neuromuscular load experience [38]. Thus, play against matched opposition may reduce match physicality, and to be less likely to induce contact injuries [9]. Notwithstanding, previous studies examining the process of development of expert basketball players revealed competing against older players as a key aspect to be more likely to become expert [39,40]. That said, playing against older or more mature players facing bigger challenges can also be a development opportunity to consider if players are robust enough to tolerate physical differences, including reduced risk of injury. Based on these findings, practitioners should select the most suitable game format, considering the individual physical, technical, tactical, and psychological development needs.

Limitations of the present study must be acknowledged. First, the participants in the current sample are slightly shorter and lighter when compared to youth basketball players of an equivalent age in other studies [3,4,6]. The degree to which maturity matching may impact physical and technical performance may vary relative to the nature of the sample. Maturity selection biases, for example, tend to be greater in more elite samples [3,4]. Second, it is important to recognize that the sample size in the current study was small and that future studies should look to consider the impact of maturity matching on a large sample [9,10,12]. Similarly, it is important to note that each athlete only completed short-duration bio-banded game formats. The impact of maturity matching may vary relative to game duration and future studies could consider playing longer matches. That said, given the current lack of knowledge regarding the effects of bio-banding in youth basketball, this study provides important starting point from which to discuss the matter and consider future research studies. Further research with a larger sample, longer game durations, players of different anthropometric attributes (height, body weight, and wingspan) should be conducted before the applicability of the current results can be generalized. Finally, it would be interesting to analyze other performance variables, such as collective tactical behavior (e.g., team synchronization), biochemical markers (e.g., creatine kinase, and testosterone), and coaches and players perceptions of unmatched competition.

Present strategy offers different challenges, and changes on environment, provide distinct physical demands and individual spatial exploration behavior according to the game format (i.e., maturity matched and un-matched). These findings may be especially useful when youth players are competing through chronological age-based competitions in which they may be more susceptible to maturation bias, particularly the less mature. Thus, our findings may help practitioners in understanding the real and potential values of talented young players during basketball training and competitions, improving the accuracy of the talent identification and recruitment processes. Furthermore, manipulate the type of opposition could be applied as a suitable strategy to individualize the prescription of training and competitive situations, considering physical and tactical development needs, and consequently encouraging the long-term developmental perspective. Moreover, this practice may be especially useful when players are going through developmental phases in which they may be more susceptible to injury risk (i.e., the pubertal growth spurt). Finally, this approach is a grouping strategy that has proposed to help counter some of the challenges presented by individual differences in growth and maturation, which not replace chronological-age competition.

## Supporting information

S1 Data Game data. (SAV)

S2 Data Physical data. (SAV)
